# Status of family planning integration to HIV care in Amhara regional state, Ethiopia

**DOI:** 10.1186/s12884-020-2838-x

**Published:** 2020-03-06

**Authors:** Zebideru Zewdie, Mezgebu Yitayal, Yigzaw Kebede, Abebaw Gebeyehu

**Affiliations:** 1 Institute of Public Health, College of Medicine and Health Sciences, University of Gondar, Gondar, Ethiopia; 2 Ethiopian Federal Ministry of Health, P.O. Box: 1234, Addis Ababa, Ethiopia; 3 Amhara National Regional State Health Bureau, Bahir Dar, Ethiopia

**Keywords:** Family planning integration, HIV positive women, Family planning use

## Abstract

**Background:**

Preventing unintended pregnancies among HIV positive women is one component of HIV prevention strategies. However, programs to prevent mother-to-child transmission (PMTCT) of HIV started in antenatal care. The objective of this study was to examine the status of family planning integration to HIV care from client and facility perspectives and identify factors associated with current family planning use.

**Methods:**

A facility-based cross-sectional study was conducted from December 2017 to April 2018. Data were coded and double entered into EPI Info version 3.5.4 and exported to STATA version 14 for analysis. Bi-variable and multivariable logistic regression analyses were conducted to assess the association of variables with the current family planning use.

**Results:**

A total of 518 HIV-positive women were included in the study. Among HIV-positive women, 35.3% had an unmet need for family planning, and 21.4% responded that their pregnancies were unwanted. About two-thirds (68.1%) of women were using a modern family planning method at the time of the study. Among women who were currently using family planning, 88.8% got the service from a family planning clinic in the same facility, and only 1.1% got the service from the HIV care unit. Women who were not knowledgeable on PMTCT (AOR 0.47, 95% CI = 0.24–0.90), divorced or separated women (AOR 0.19, 95% CI = 0.10–0.37) and women in the age group of 25–34 years (AOR 0.42, 95% CI = 0.20–0.88) and 35–49 years (AOR 0.41, 95% CI = 0.17–0.99) were less likely to use modern family planning methods compared with those women who were knowledgeable, married and women in the age group of 15–24 years. Besides, women with higher income (AOR 2.12, 95% CI = 1.26–3.57) were more likely to use modern family planning methods compared with women with lower incomes.

**Conclusion:**

This study indicated that there is a high unmet need for family planning among HIV-positive women and low family planning services integration in the PMTCT/ART clinics. Efforts should be strengthened to tackle the factors which hinder the use of modern family planning and improve family planning service integration.

## Background

Women of childbearing age account for more than half of the world’s Human Immunodeficiency Virus (HIV) cases and unintended pregnancy is high among HIV positive women [1]. Globally, nearly 1700 infants become infected with HIV every day and 90% of these new infections are acquired through mother-to-child transmission (MTCT). The uses of family planning methods were among the strategies to prevent HIV positive birth [1]. This indicates that strengthening family planning services to HIV positive women gives an added value to prevent MTCT.

Globally, 222 million women either want to delay or avoid their pregnancy but they are not using modern contraceptive methods. This includes women who are HIV positive and those at risk of HIV [2–4]. Added to this, without appropriate prevention of mother to child transmission (PMTCT) interventions, about 50% of those infants born with HIV will die before their second birthdays [5–7]. Global commitments to eliminate new pediatric HIV infections recognized that preventing unintended pregnancies in HIV positive women is an essential HIV prevention strategy [8]. Therefore, preventing unintended pregnancies among women living with or at-risk of contracting HIV is a key component of the global HIV prevention strategy [8]. Family planning can also help achieve HIV prevention goals and improve maternal and child health outcomes [2].

Integrating family planning and HIV services is important to get information and services for both family planning and HIV care. Ensuring access to family planning options in PMTCT services saves time, increases uptake, improves the productivity of nurses, and decreases patient waiting times [9]. Additionally, this strategy promotes a one-stop shopping service that is user-friendly that improves the health outcomes of mothers and children [10]. There are different modalities of service integrations in different settings [11, 12]. These include the provision of family planning within HIV testing, care, or treatment sites within PMTCT services, or within peer-outreach and community-based and home-based programs.

Ethiopia is among the 21 global priority countries with the highest number of pregnant women living with HIV who need PMTCT services [13]. These 21 countries accounted for 90% of the total number of pregnant women living with HIV that need services to prevent MTCT of HIV. In Ethiopia, there were 29,630 HIV positive mothers needing PMTCT services in 2017 and 8653 were from the Amhara region [14]. The adult HIV prevalence was also estimated to be 1.2% in 2014 [15, 16]. The report also showed that the number of pregnancies among HIV positive mothers of the Amhara Region in 2014/2015 was 61.2% [17].

According to the 2013/2014 report of the Ethiopian HIV Prevention and Control Office (HAPCO), 18.8% of pregnant women on ART reported that their pregnancy was unintended [18]. The Federal Ministry of Health of Ethiopia has started integrating family planning with ART unit and HIV testing and counseling (HTC), and PMTCT within maternal, newborn and child health services since 2015. However, the programs to prevent MTCT of HIV continue to be executed as a narrow set of interventions that initiates in antenatal care. Besides, limited studies are observed on family planning integration in Ethiopia as well as the Amhara region. Therefore, this study aimed to examine the status of family planning integration to HIV care from client and facility perspectives and identify factors associated with current family planning use.

## Methods

### Study design and area

A cross-sectional study design was employed to assess the status of family planning integration to HIV care from client and facility perspectives in three referral hospitals (Gondar Referral Hospital, Dessie Referral Hospital, and Debre Birhan Referral Hospital) and three health centers (Gondar Town Health Center, Dessie Health Center and Debre Birhan Health Center) in Amhara region from December 2017 to April 2018. Amhara region is located in the northwestern part of Ethiopia.

### Study population and sampling

All HIV-positive women who gave birth during the last 6 weeks to 24 months and who were on PMTCT follow up during the study period were included in the study. Also, facility audits in all selected health facilities were done to assess the availability of family planning services. However, all HIV-positive women who were seriously sick (women who were unable to communicate due to illness), who gave birth within the last 6 weeks, and who were pregnant were excluded from the study.

The sample size was determined using a single population proportion formula and calculated using EPI-info version 7.2.1.0 software. The sample was determined based on prevalence of unmet need for family planning which is 18.8% among women living with HIV [18] which is a proxy indicator to see family planning and HIV integration status, 5% margin of error, 95% confidence interval and design effect of 2, and 10% non-response rate. Hence, the final sample size of the study was 517 HIV-positive women.

A multi-stage sampling technique with simple random sampling at each stage was employed. At the first stage, three referral hospitals and three health centers were selected by using a simple random sampling technique. In the second stage, HIV positive women who fulfilled the inclusion criteria were selected using a systematic random sampling technique from the PMTCT unit. The PMTCT registers were the sampling units and HIV-positive women on PMTCT follow up were the study participants. The number of study participants from each selected health facility was determined using proportional to population size calculation.

### Data collection procedures

A structured questionnaire was used to collect the data. The structured questionnaire was prepared in English and translated to Amharic and then back to English to keep its consistency. The questionnaire was developed by reviewing journal articles, WHO and MOH PMTCT guidelines, and mother-baby pair cohort registers [19–21] (Supplementary file 1). It consisted of the dependent variables: family planning integration status and current use of family planning; and independent variables such as personal factors (socio-demographic factor, maternal education, monthly income), reproductive and related factors (number of pregnancy, and number of ANC visits); knowledge about HIV testing and counseling, MTCT, and PMTCT; institutional factors (privacy of ART room, method mix for family planning); social factors (stigma and discrimination, partner disclosure); and desire to have children, unintended pregnancies, partner desire to have children, previous use of a contraceptive method and unmet need of family planning.

Twelve data collectors and four supervisors were recruited to administer the questionnaire using exit interviews. Exit interviews were conducted with a pre-tested structured questionnaire after taking informed consent from the study participants.

### Data processing and analysis

Statistical analysis was undertaken using STATA version 14. Frequencies, proportions, and means were used to describe the data. A bivariate logistic regression analysis was carried out to see the association of socio-demographic factors, maternal and reproductive factors, and knowledge of MTCT of HIV on the current use of family planning. Variables associated with the outcome variable during bivariate logistic regression at *p*-value ≤0.2 were included in the multiple logistic regression models, using backward fitting. Variables persisted to be associated with the outcome variable at *P* ≤ 0.05 were considered to be significantly associated with the outcome variable.

The reliability of the tools was measured using Cronbach’s alpha value and scales with a value above 0.7 were believed as reliable. Overall model fitness was measured using the log-likelihood ratio test. Based on the statistical significance of the probability of model chi-square less than 0.05 was supported the model to be a good fit. Hosmer and Lemeshow test goodness of fit were also used as a statistical value of more than 0.05 to characterize a logistic regression model as a better fit. A Wald test was used to test the statistical significance of the relationship between current family planning use and individual independent variables in the model.

Family planning integration was defined as the provision of family planning services (short and long-acting family planning methods in addition to condom) within HIV testing, care, or treatment sites, and within the PMTCT unit. Unmet need for family planning was also defined as the proportion of women with no desire to become pregnant immediately before the most recent pregnancy but who are not using any method currently.

### Ethical considerations

The University of Gondar Ethics Review Board approved the research proposal. A support letter was secured from the Amhara National Regional Health Bureau. Written informed consent was obtained from all research participants after the explanation of study objectives. The aims, benefits and the time it takes to complete the questionnaire were explained to the participants. Besides confidentiality, anonymity and their right to refuse from participation in the study were explained to them.

## Results

### Socio-demographic characteristics of the study participants

Five hundred eighteen HIV positive women participated in the study with a response rate of 100%. The estimated sample size was 517 but one additional participant was included in the study due to her presence during the study period. Among the 518 HIV positive women interviewed, 389 (75.1%) were orthodox Christians, 410 (79.2%) were married, 113(21.8%) unable to read and write and 253(48.8%) were housewives. The majority (66.4%) of the respondents were in the age group of 25–34 years. The median age was 30 years (IQR: 27–33 years), and the median family monthly income was 1500 Birr. Three hundred eleven (60%) HIV-positive women had one to two pregnancies at the time of study and 71.2% of the respondents’ youngest child age was 3–12 months (Table 1).

**Table 1:** Socio-demographic characteristics of study participants in Amhara Regional State, Ethiopia, 2018

Variables	Frequency (***n*** = 518)	Percentage (%)
Age (Years)		
18–24	57	11.0
25–34	344	66.4
35–49	117	22.6
Religion		
Orthodox	389	75.1
Muslim	110	21.2
Protestant	19	3.7
Marital Status		
Single	30	5.8
Married	410	79.2
Divorced/Separated	68	13.1
Widowed	10	1.9
Education		
Unable to read and write	113	21.8
Able to read and write	72	13.9
Primary education	129	24.9
Secondary education	111	21.4
Diploma	66	12.8
Degree	27	5.2
Occupation		
Housewife	253	48.8
Merchant	96	18.5
Government employee	86	16.6
Day laborer	67	12.9
Commercial Sex Worker	9	1.8
Farmer	7	1.4
Ethnicity		
Amhara	463	89.4
Tigre	28	5.4
Oromo	27	5.2
Monthly family income (*Ethiopian Birr)		
0–1500 Birr	269	51.9
1501–3000 Birr	154	29.7
> 3000 Birr	95	18.4
Number of pregnancies		
1–2 times	311	60.0
3–4 times	175	33.8
> 5 times	32	6.2
Age of youngest child (months)		
2 months	51	9.8
3–12 months	369	71.2
13–24 months	98	19.0

□ *27.21Ethiopian Birr = 1 USD*

### Fertility desires and family planning history

About 85.9% of HIV-positive women gave birth after knowing their HIV status and 70.8% HIV-positive women had used the family planning method before their last pregnancy. Overall, 33.2% of the respondents who already had children had the desire to have more children. Among HIV-positive women, 49.4% intended to have another child after 2 years. For nearly two-thirds (59.9%) of the study participants, the reason to have more children was to replace themselves. Among HIV-positive women, 35.3% had an unmet need for family planning, 21.4% ever had an unwanted pregnancy and 12.9% ever had induced abortion (Table 2).

**Table 2:** Fertility desires and unplanned pregnancies among HIV-positive women in Amhara Regional State, Ethiopia, 2018

Variables	Frequency (***n*** = 518)	Percentage (%)
Gave birth after knowing HIV status		
Yes	445	85.9
No	73	14.1
Use of family planning before your last pregnancy		
Yes	367	70.8
No	151	29.2
Plan to be pregnant again		
Yes	172	33.2
No	309	59.7
Do not know	37	7.1
Reason to give birth (*n* = 172)		
To replace myself	103	59.9
My partner wants	51	29.7
To hide from people and avoid stigma and discrimination	3	1.7
To hide my HIV status from my partner	2	1.2
Do not know	4	2.3
Others	9	5.2
Preference to have children (*n* = 172)		
Within two year	19	11.1
After two year	85	49.4
When I feel healthy	22	12.8
When my CD4 is high	22	12.8
Do not know	24	13.9
Number of children do you intend to have in the future (*n* = 172)		
One	56	32.6
Two	52	30.2
Three	40	23.3
Don’t know	24	13.9
Ever had unwanted pregnancy		
Yes	111	21.4
No	407	78.6
Ever had Induced abortion		
Yes	67	12.9
No	451	87.1
Not have a plan to be pregnant again (*n* = 309)		
Not using FP currently (Unmet Need of FP)	109	35.3
Using FP currently	200	64.7

### Family planning integration to HIV care and current use of family planning methods

From 518 HIV-positive women, the majority (96.5%) discussed family planning with their health care provider in the current visit and 68.1% were currently using a modern family planning method. The most frequent methods of contraception among family planning users were implant (35.9), followed by injectable (32.6%), IUCD (14.2%), condom (8.2%) and 8.0% used Pills. Dual method use (condom and other methods such as pills, injectable, and IUCD) was reported by only 2.8%. Among women who currently used family planning, 41.1% preferred to get the service at ART/PMTCT clinic and 52.1% preferred to get the service in the family planning unit. However, among women who currently used family planning, the majority (88.8%) got the service from the family planning clinic in the same facility and only 1.1% got the service from PMTCT/ART unit (Table 3).

**Table 3:** Family planning integration and current family planning use among HIV positive women in Amhara Regional State, Ethiopia, 2018

Variables	Frequency (***n*** = 518)	Percentage (%)
Discussed about family planning with the health care provider in the current visit?		
Yes	500	96.5
No	18	3.5
Using family planning method currently		
Yes	353	68.1
No	165	31.9
Methods of Contraception used (*n* = 353)		
Implant	127	35.9
Injectable	115	32.6
IUCD	50	14.2
Male condom	29	8.2
Pills	28	8.0
Others	4	1.1
Dual method (condom and other method) (*n* = 353)		
Yes	10	2.8
No	343	97.2
Reason for choosing this contraceptive. (*n* = 353)		
Because it suits to my health	239	67.7
Health professionals Preference	62	17.6
From my friends experience	50	14.1
Others	2	0.6
Reason for using family planning method (*n* = 353)		
To space birth	190	53.8
To stop birth	107	30.3
To limit the number of child	56	15.9
Place of getting family planning service (*n* = 353)		
From Family planning clinic in the same facility	326	92.4
Family planning clinic in another service	23	6.5
From PMTCT/ART unit	4	1.1
Place of preference to get family planning service (*n* = 353)		
In family planning unit	191	54.1
At ART/PMTCT clinic	145	41.1
Private clinic	17	4.8
Reason for not using family planning method (*n* = 165)		
I have no partner	78	47.3
I am using condom	30	18.2
I want to give birth	25	15.2
Fear of side effects	16	9.7
My partner doesn’t agree	10	6.1
Others	6	3.6

### Family planning service audit

In all selected health facilities there were separate service delivery points for family planning, PMTCT, and ART units. Two of the six health facilities had a poster in PMTCT and ART unit advertising family planning services. There was a limited choice of family planning methods available in those ART/PMTCT clinics providing family planning services. The health care workers of two ART clinics were trained on family planning. Among the six facilities, only two health facilities were providing family planning services other than male condoms at the HIV clinic (PMTCT/ART). Only oral contraceptives and condoms were available in those ART/PMTCT clinics providing family planning services.

### Factors associated with current use of modern family planning methods

The current use of modern family planning was significantly associated with maternal age, marital status, family income, and knowledge of MTCT. Women in the age group of 25–34 years were 42% (AOR 0.42, 95% CI = 0.20–0.88) and women in the group of 35–49 years were 41% (AOR 0.41, 95% CI = 0.17–0.99) less likely to use modern family planning methods compared with women in the age group of 15–24 years. Divorced or separated women were 19% (AOR 0.19, 95% CI 0.10–0.37) less likely to use modern family planning methods compared with married women. Women with higher income were 2.12 (AOR 2.12, 95% CI = 1.26–3.57) times more likely to use modern family planning methods compared with women with lower income, and women who were not knowledgeable on PMTCT were 47% (AOR 0.47, 95% CI = 0.24–0.90) less likely to use modern family planning methods than those who were knowledgeable (Table 4).

**Table 4:** Crude and multivariate logistic regression models for factors associated with current family planning use (*n* = 518), 2018

Explanatory Variable	Current use of modern FP		Crude OR (95% CI)	Adjusted OR (95% CI)	P-value
	Yes	No			
Age					
15–24	42	15	1	1	
25–34	232	112	0.73 (0.39, 1.39)	**0.42 (0.20, 0.88)**	**0.022**
35–49	79	38	0.74 (0.36, 1.50)	**0.41 (0.17, 0.99)**	**0.048**
Religion					
Orthodox	267	122	1	1	
Protestant	10	9	0.50 (0.20, 1.28)	0.37 (0.12, 1.07)	0.069
Muslim	76	34	1.02 (0.64, 1.61)	1.12 (0.66, 1.90)	0.651
Marital Status					
Married	302	108	1	1	
Single	22	8	0.98 (0.42, 2.27)	1.39 (0.53, 3.62)	0.494
Widowed	5	5	0.35 (0.10, 1.25)	0.52 (0.11, 2.34)	0.401
Divorced/Separated	24	44	**0.19 (0.11, 0.33)**	**0.19 (0.10, 0.37)**	**0.000**
Education level					
Unable to read & write	75	38	1	1	
Able to read & write	135	66	1.03 (0.63, 1.68)	0.88 (0.50, 1.55)	0.677
Secondary and above	143	61	1.18 (0.72, 1.94)	**0.50 (0.26, 0.95)**	**0.037**
Occupation					
Government employee	69	17	1	1	
Merchant	55	41	**0.33 (0.16, 0.64)**	**0.26 (0.12, 0.59)**	**0.001**
Housewife	186	74	0.61 (0.34, 1.12)	**0.44 (0.20, 0.93)**	**0.032**
Day laborer	43	33	**0.32 (0.15, 0.64)**	**0.37 (0.15, 0.91)**	**0.031**
Ethnicity					
Amhara	318	145	1	1	
Oromo	19	8	1.08 (0.46, 2.53)	1.42 (0.53, 3.83)	0.482
Tigrie	16	12	0.60 (0.28, 1.31)	0.92 (0.37, 2.42)	0.881
Monthly family income (Ethiopian Birr*)					
≤ 1500 Birr	164	105	1	1	
1501–3000 Birr	118	36	**2.09 (1.34, 3.27)**	**2.12 (1.26, 3.57)**	**0.005**
> 3000 Birr	71	24	1.89 (1.12, 3.19	1.80 (0.96, 3.36)	0.064
No of Pregnancies? (Gravida)					
1–2 times	207	104	1	1	
3–4 times	128	47	1.36 (0.90, 2.05)	1.48 (0.92, 2.39)	0.100
> 5 times	18	14	0.64 (0.30, 1.34)	0.58 (0.24, 1.40)	0.232
Knowledge about PMTCT					
Knowledgeable	324	142	1	1	
Not Knowledgeable	29	23	**0.55 (0.30, 0.98)**	**0.47 (0.24, 0.90)**	**0.024**
Place of birth youngest child					
In this hospital	305	142	1	1	
In another hospital	45	18	1.16 (0.65,2.08)	1.39 (0.72, 2.67)	0.313
Deliver at home	3	5	0.27 (0.06, 1.18)	0.24 (0.04, 1.24)	0.089
Disclosure status					
Yes	313	132	1	1	
No	40	33	**0.51 (0.30, 0.84)**	0.81 (0.43, 1.51)	0.516

## Discussion

In this study, 96.5% had discussed family planning with their health care provider in the current visit and 68.1% were currently using a modern family planning method. The finding was higher than the findings of Ghana (42.6%) [22], Tanzania (54%) [23], Malawi (51.2%) [24], and Horro Guduru Wollega zone, Ethiopia (46.6%) [25]. However, the current contraceptive use among respondents in this study is similar to studies among HIV-positive women in Cambodia (68.5%), Zambia (69%), Uganda (58%), and Dire Dawa, Ethiopia (69%) [26–29].

In this study, the most frequent method of contraception among HIV-positive women was Implant (35.9%), followed by injectable (32.6%). However, only 8.2% of the study participants were using condom. Even though the use of condoms is essential among HIV-positive women, the study revealed that the use of condoms and dual method use among respondents is low compared with similar studies in which show 79.6% male condom use in Ghana [22], and 42.9% in Dire Dawa, Ethiopia [27]. This may be explained by the current study uses only women as study population and women might not directly control condom use. This will be a great concern for the risk of HIV transmission.

This study reveals that a high proportion of women use implant among family planning users which is higher compared with the findings of other studies conducted in Ghana (7.1%), Tanzania (13.3%), and Zambia (8%) [22–23,28]. This may be explained by the availability of service at a lower health facility level (the service is provided by a health extension worker at a health post level in Ethiopia).

The study reveals that, despite being HIV-positive, 33.2% of the respondents who already had children had the desire to have more children which is much higher than the finding of a study in Cambodia (7.3%) [26]. The finding is consistent with the study conducted in Dire Dawa, Ethiopia where 32% of respondents had the desire to have more children [27]. The study also reveals that 60% of HIV-positive women had one to two pregnancies at the time of study and 85.9% gave birth after knowing their HIV status in this study. Low risk of MTCT after ARV may favor this outcome among HIV-positive women.

The integration of family planning and HIV services greatly helps in supporting the implementation of prong two at all levels of program and health service management. Addressing women’s reproductive health needs holistically strengthens the impact of both family planning and HIV services [10]. The integration of family planning services to HIV services can increase access and uptake [2, 9]. Family planning integration has increased the use of family planning by 12.9% in Kenya [30]. But, in this study, only 1.1% got the service from PMTCT/ART unit. The facility audit also showed that among the six facilities, only two health facilities were provided with family planning services other than male condoms at the HIV clinic (ART/PMTCT unit). There was a limited choice of family planning methods available in those ART/PMTCT clinics providing family planning services. Health workers who were working in only two ART clinics had been trained in family planning. This shows the integration of services was poor. This finding is in line with the findings of a study conducted in five countries of Africa (Ethiopia, Kenya, Rwanda, South Africa, and Uganda) [31] on integration of family planning to HIV care, which shows that most ART clinics had only one or two providers on duty and many of these providers indicated that they did not have family planning training. This study also has a similar finding with a study conducted in Uganda which showed that ART clinic did not have contraceptives onsite [32]. Similar studies conducted in Ethiopia and South Africa showed the presence of weak integration of family planning services to chronic HIV care [27, 33]. A study conducted in Kenya showed that 73% of women and 71% of men thought that they or their partner would be more likely to use family planning if the services were offered at the HIV clinic [34]. Besides, a study in Zambia revealed that most respondents said they would access family planning services had it been available within the ART clinic [28].

This study indicates that a high proportion of women with no desire to be pregnant were not using any modern method of contraception and there was no method mix at ART/PMTCT unit in those facilities that integrate the service. This might have been a factor for low uptake of modern family planning and hence strengthening the integration of family planning services in the Adult HIV clinic and PMTCT clinic is important.

The unmet need for family planning among HIV-positive women in this study is higher compared to the unmet need among the general population in Amhara Region, Ethiopia (17%) [35]. This is also supported by a study conducted in Dire Dawa, Ethiopia which shows a high unmet need for family planning (36%) among women living with HIV [27]. The finding is comparatively higher than the findings reported from Kenya (20%) [31], Ghana 27.8% [22], Western Ethiopia (15.4%) [36], and Hawassa city Ethiopia (19.1) [37]. This study also indicates that 21.4% of participants ever had an unwanted pregnancy and 12.9% ever had induced abortion.

In this study maternal age, marital status, family income, and knowledge about MTCT were strongly associated with the current use of contraception. Women in the age group of 25–34 years and women in the group of 35–49 years were less likely to use modern family planning methods compared with women in the age group of 15–24 years. This is consistent with the findings of studies conducted in Malawi [38], and Ethiopia [39]. Divorced or separated women were less likely to use modern family planning methods compared with married women. This is in line with studies done in Kenya [40], Nigeria [41], and Ethiopia [39]. This study reveals that women with higher income were more likely to use modern family planning methods compared with women with lower income and women who were not knowledgeable on MTCT were less likely to use modern family planning methods than those who were knowledgeable. Similar findings were found from studies conducted in Nigeria [41], and Ethiopia [39]. This might be due to the effect of women’s financial capacity and knowledge about MTCT which may enable women decision making autonomy on the couple’s family planning use. This highlights the need to devise income-generating strategies and increase the knowledge of women about MTCT of HIV.

Limitations of this study, due to the cross-sectional nature of the data, cause and effect relationship between the dependent and independent factors could be weak; and social desirability bias may not be avoidable even though data collectors were trained. Besides, current contraceptive use was based on self-report by the women who might have reported what was perceived as socially acceptable or correct, which would have overestimated the estimates obtained in this study. Despite these limitations, the data being gathered have a better description of family planning among HIV positive women.

## Conclusion

The study shows that there is a high unmet need for family planning among HIV-positive women and low integration of family planning services in PMTCT/ART clinics. Knowledge about MTCT of HIV, family income, marital status, and maternal age are predictors of current contraceptive use. Reducing the unmet need for modern family planning methods by improving the provision of family planning services to HIV-positive women through the integration of family planning to HIV care and increasing awareness of HIV positive women about PMTCT is recommended for prevention of mother to child transmission of HIV.

## Supplementary information

**Additional file 1:** English Version Questionnaires

## Data Availability

The datasets used during the current study are available from the corresponding author on reasonable request.

## Abbreviations

AOR: Adjusted odds ratio
ART: Antiretroviral therapy
CI: Confidence interval
FP: Family planning
HCT: HIV counseling and testing
HIV: Human immunodeficiency virus
MTCT: Mother-to-child transmission
OR: Odds ratio
PMTCT: Prevention of mother-to-child transmission
