# Supplementary material for: Status of family planning integration to HIV care in Amhara regional state, Ethiopia
Source: BMC Pregnancy Childbirth. 2020 Mar 6;20:145. doi: 10.1186/s12884-020-2838-x (PMC7059673; doi:10.1186/s12884-020-2838-x)
Supplement: Supplementary file 1 — Additional file 1: English Version Questionnaires [file 12884_2020_2838_MOESM1_ESM.doc]

English Version Questionnaires

**Information Sheet and Consent Form for Status of family planning integration to HIV care in Amhara Regional State, Ethiopia**

# My name is ___________, I am a nurse/midwifes working other health facilities now I am collecting data for the research being conducted among HIV positive women to assess status of family planning integration to HIV care. The purpose of this study is to assess the status of family planning integration to HIV care in Amhara Regional State, Ethiopia. The findings of this study will contribute to prevent and control HIV infection by assessing family planning service utilization among HIV positive women and proposing possible solution to theoretical and practical problems that have been encountered in the process of service provision.

# The information collected from this research project will kept confidential and information about you that will be collected by this study will be stored in a file, without your name, but a code number assigned to it. It will not be revealed to anyone except the principal investigator and will be kept locked with key. If you agree to be interviewed, you can still withdraw at any time you want to do so. Your refusal will not affect you from getting any kind of health related service. The usual care will be provided whether you participate in this study or not. The method of the interviewees’ selection is random.

**Informed Consent Form**

# I, here, undersigned to participate on currently conducted research on the title “Status of family planning integration to HIV care in Amhara Regional State, Ethiopia”. The finding of study will be used to generate information for program expansion and designing strategies for MTCT prevention in the region and elsewhere with similar characteristics.

I understand that I have the right to withdraw from this interview at any time and the usual care will be provided whether I participate in this study or not. Furthermore, I understand that the information discussed in this interview is strictly confidential and there will not be any disclosure of names or identity. The research will involve an interview which will take about 20-30 minutes.

I hereby volunteer to participate in the study and give the research team permission to contact me for the purpose of collecting data.

Participant’s signature:_________________________ date:_____________

Interviewer’s signature: ________________________ date:_________

Checked by supervisor signature: ________________date________

English Version Questionnaires University of Gondar College of Medicine and Health sciences Institute of Public Health

Questionnaires for exit interview of HIV positive women on the assessment of the Status of family planning integration to HIV care

Region _______Zone _________Health facility____________ Respondent Code____________

Section I- Socio-Demographic and Economic Characteristics

| No. | Questions | Response coding categories | Skip this Q for |
| --- | --- | --- | --- |
| 101 | Age (in completed years) | ______________ years |  |
| 102 | Religion | 1. Orthodox  2. Protestant  3. Catholic  4. Muslim  88.Others(specify)--------------- |  |
| 103 | What is your marital status? | 1. Married  2. Single  3. Widowed  4. Separated  5. Divorced | If the response is 1 go to Q 104 else go to Q105 |
| 104 | If married are you currently living with your partner? | 1. Yes  2. No |  |
| 105 | Education | 1. Unable to read and write  2. Able to read and write  3. Primary education  5. Secondary education  6. Diploma  7. Degree  88. Other specify------------ |  |
| 106 | Occupation | 1. Government employee  2. Merchant  3. Housewife  4. Student  5. Day laborer  6. Farmer  7. Commercial Sex Worker  88. Others (specify_________ |  |
| 107 | What is your average house hold income per month? | ---------- birr |  |
| 108 | Ethnicity | 1. Amhara  2. Oromo  3. Tgre  4. Agew  5. Kimant  88. Other (specify) -------- |  |

Section II – Reproductive and Family planning related question

| No. | Questions | Response coding categories | Skip this Q for |
| --- | --- | --- | --- |
| 201 | Gravidity (number of pregnancy) | _______________( put in number) |  |
| 202 | How many live births have you had?  (Number of children) | _______________( put in number) |  |
| 203 | Where did you give birth of the current child? | 1. In this hospital  2. In another hospital offering PMTCT  3. In another hospital, don’t know if PMTCT is offered.  4. Deliver in a TBA”s place  5. Deliver at home under supervision of relatives |  |
| 204 | How many antenatal care visits do you have in the previous pregnancy? | 1. One 2. Two 3. Three 4. Four and above |  |
| 205 | Did you give birth after you knew your HIV status? | 1.Yes  2. No  3.Refuse to tell |  |
| 206 | Was your last pregnancy wanted/timed? | 1.Yes  2. No  3. Refuse to tell  99. Don’t know |  |
| 207 | Did you use family planning before your last pregnancy? | 1. Yes 2. No | If the response is yes go to Q 208 else go to Q210 |
| 208 | Where did you get your FP? | 1. From Family planning clinic in the same facility  2. Family planning clinic in another service  3. From PMTCT/ART unit  88. Other specify ---------  99. Don’t know |  |
| 209 | What type of contraceptive method were you using? | 1. Male Condom  2. Pills  3. Injectable  4. Implant  5.IUCD  88. Other specify ---------  99. Don’t know |  |
| 210 | Have you discussed about family planning with your health care provider in the current visit? | 1. Yes  2. No |  |
| 211 | Do you have a plan to be pregnant again? | 1.Yes  2. No  3. Refuse to tell  99. Don’t know | If the response is yes go to Q 212 else go to Q 215 |
| 212 | Why do you need to give birth? | 1. To hide from people  2. To avoid stigma and discrimination  3. To replace my heredity  4. To hide my HIV status from my  partner  5. My partner wants  88. Other specify, ----------------------  99. Don’t know |  |
| 213 | When you prefer to have children? | 1. Within two year  2. After two year  3. When I feel healthy  4. When my CD4 is high  88. Other specifies -----------------------  99. Do not know |  |
| 214 | How many children do you intend to have in the future? | 1. One  2. Two  3. Three  88. Other specify______  99. Don’t know |  |
| 215 | Are you using family planning method currently? | 1.Yes  2. No | If the response is yes go to Q 215 else go to Q 219 |
| 216 | If you using family planning method currently, what type of method are you using?  **(More than one response is possible)** | 1. Male Condom  2. Pills  3. Injectable  4. Implant  5. IUCD  88. Other, specify___________ |  |
| 217 | Why you choose to use this contraceptive? | 1. Health professionals Preference  2. Because it suits to my health  3. From my friends experience  88. Other, specify____________ |  |
| 218 | Why do you want to use family planning method? | 1. To space birth  2. To limit the number of child  3. To stop birth  88. Other specify---------------- |  |
| 219 | Where do you prefer to get the service? | 1. At ART/PMTCT clinic  2. In FP unit  3. Private clinic  88. Other specify-------------- |  |
| 220 | Why do not you use family planning method? | 1. Fear of side effects  2. My partner doesn’t agree  3. I have no partner  4. I want to give birth  5. I am using condom  88. Other ,specify-------------------- |  |
| 221 | Have you ever had unwanted pregnancy? | 1. Yes 2. No |  |
| 222 | Have you ever had induced abortion? | 1. Yes 2. No |  |

Section III – knowledge and attitude of HIV positive women towards MTCT and PMTCT of HIV related questions

| No | Questions | Response coding categories | Skip to |
| --- | --- | --- | --- |
| 301 | What are the modes of HIV transmission? ( Do not read, Circle what she says) | 1. Sexual intercourse  2. Blood and blood products  3. MTCT  4. Injections and blades  77. No response  99. I do not know |  |
| 302 | What are the methods to prevent one from being infected with HIV? ( Do not read, Circle what she says) | 1. Abstinence  2. Faithful one to one relationship  3. Use of condom  77. No response  99. I do not know |  |
| 303 | Have you ever heard about PMTCT before? | 1.Yes  2. No | If the response is yes go to Q 304 else go to Q 305 |
| 304 | Where did you hear about it? (Do not read the alternatives. More than one response is possible) | 1. Friends  2. Relatives  3. Health professional  4. Radio /TV  88. Other, specify_________ |  |
| 305 | Can HIV be transmitted from Mother to Baby? | 1.yes  2. No  77. No response  99. I do not know |  |
| 306 | Can HIV be transmitted from Mother to Baby during pregnancy? | 1.yes  2. No  77. No response  99. I do not know |  |
| 307 | Can HIV be transmitted from Mother to Baby during delivery? | 1. yes  2. No  77. No response  99. I do not know |  |
| 308 | Can HIV be transmitted from Mother to Baby during breastfeeding? | 1.yes  2. No  77. No response  99. I do not know |  |
| 309 | Are you aware of interventions that can prevent MTCT of HIV? | 1.yes  2. No  77. No response  99. I do not know | If the response is yes go to Q 310 else go to Q 311 |
| 310 | What are they?  (More than one response is possible) | 1. Use of ARV drugs for mother and NVP prophylaxis for the infant  2. Exclusive breast feeding.  3. DNA/PCR test for child  99. I do not know |  |
| 311 | Would you tell your partner the result of your HIV test ? | 1. Yes  2. No  77. No response  99. I don’t know |  |
| 312 | Do you think that HIV counseling and testing is important for pregnant women? | 1. Yes  2. No  77. No response  99 I don’t know |  |
| 313 | If your sister, relative or friend become pregnant would you advise them that it is good to be tested for HIV? | 1. Yes  2. No  77. No response |  |
| 314 | Would you recommend HIV testing to anybody? | 1. Yes  2. No  77. No response |  |

End of the questionnaires. Thank you!!

Name of data collector _____________________Date of data collection __________________ Signature ___________________________________

**Facility audit guide questions**

**Facility name ………………………………………City……………………………………………….**

**Health Facilities included in the Facility Audit (health facility guide question)**

| No | Questions | Response coding categories | Comment |
| --- | --- | --- | --- |
|  | **FP Services/Methods Available** |  |  |
| 1 | Posters advertising FP service | 1.Yes  2. No |  |
| 2 | FP counseling provided | 1.Yes  2.No |  |
| 3 | FP methods available in the health facility | 1 Male condom  2. Oral contraceptives  3. Injectable  4. Implants  5. IUDs  6. Natural FP  7.Tubal ligation |  |
| 4 | Status/Availability of FP Methods at PMTCT/ART unit during Visits | 1.Combined oral  2.Contraceptive pills  3. Progestin only pills  4. Injectable  5. Implant  6. Male condom  7. Female condom  8. IUCD  9. Spermicide  10.Tubal ligation |  |
| 5 | FP poster displayed in HIV clinic (PMTCT/ART clinic) | 1.Yes  2.No |  |
| 6 | HIV services available | 1. HIV counseling and  2. Testing service  3.PMTCT  4. ART  5.Ongoing counseling  6.Tx of opportunistic infections |  |
| 7 | Providers trained in FP at HIV clinic (PMTCT/ART clinic) | 1.Yes  2.No |  |
| 8 | HIV clinic provides FP services (other than male condoms) | 1.Yes  2.No |  |
| 9 | FP services and methods available in HIV clinic (PMTCT/ART unit) | 1. FP counseling  2. Male condoms  3. Oral contraceptives  4. Injectable  5. IUCD |  |
